# Hantavirus antibody seroprevalence and risk factors among adults in West Kazakhstan, 2023

**DOI:** 10.3389/fpubh.2024.1519117

**Published:** 2025-01-16

**Authors:** Ulyana Gubareva, Roberta Horth, Dilyara Nabirova, Nur Tukhanova, Elmira Utegenova, Zhanna Shapiyeva, Zangar Turliyev, Nazym Tleumbetova, Nurbek Maykanov, Manar Smagul, Alan L. Landay, Gavin Cloherty, Francisco Averhoff, Edmond F. Maes

**Affiliations:** ^1^Scientific and Practical Center for Sanitary-Epidemiological Expertise and Monitoring, Almaty, Kazakhstan; ^2^Central Asia Field Epidemiology Training Program, Asfendiyarov Kazakh National Medical University, Almaty, Kazakhstan; ^3^Project Implementation Unit of the Global Fund to Fight AIDS, Tuberculosis and Malaria in Kazakhstan, Almaty, Kazakhstan; ^4^Division of Global Health Protection in Central Asia, United States Centers for Disease Control and Prevention, Almaty, Kazakhstan; ^5^S. Asfendiyarov Kazakh National Medical University, Almaty, Kazakhstan; ^6^M. Aikimbayev’s National Scientific Center of Especially Dangerous Infections, Almaty, Kazakhstan; ^7^International Institution of Postgraduate Education, Almaty, Kazakhstan; ^8^Reference Laboratory for the Control of Especially Dangerous Infections, Almaty, Kazakhstan; ^9^Ural Anti-Plague Station, Uralsk, Kazakhstan; ^10^National Center for Public Health, Astana, Kazakhstan; ^11^University of Texas Medical Center, Galveston, TX, United States; ^12^Infectious Diseases Research, Abbott Diagnostics, Abbott Park, IL, United States; ^13^Abbott Pandemic Defense Coalition (APDC), Abbott Park, IL, United States; ^14^Department of Global Health, Emory Rollins School of Public Health, Atlanta, GA, United States

**Keywords:** hantavirus, hemorrhagic fever renal syndrome, HFRS, hantavirus infection, rodent-borne diseases, West Kazakhstan

## Abstract

**Background:**

Orthohantaviruses (also known as hantaviruses) are pathogens, primarily transmitted by rodents, that can cause hemorrhagic fever with renal syndrome (HFRS). In endemic regions of Kazakhstan, no confirmed HFRS cases were detected between 2020 and 2022 raising concerns about detection. Estimate antibody seroprevalence for hantaviruses and identify associated risk factors among high-risk adults in western Kazakhstan in 2023.

**Methods:**

In this cross-sectional study, adults were randomly sampled from public clinic registries in 14 villages in West Kazakhstan during June–July 2023. We interviewed 921 participants and collected serum samples which were tested for presence of hantavirus specific IgG antibodies using enzyme-linked immunosorbent assay (ELISA). Socio-demographic, clinical characteristics, and residential risk-factor data were self-reported. We assessed factors associated with seropositivity using multivariable Poisson regression, adjusting for key variables such as age and gender.

**Results:**

Among 921 participants, 63.0% were female, median age was 53 years, 72.0% resided in single houses and 38.0% reported encounters with rodents. Among 921 participants we found 3.1% (*n* = 28) hantavirus seroprevalence (95% confidence interval [CI]: 2.1–4.3). No seropositive participants had prior hospitalization or symptoms consistent with hantavirus. Three seronegative participants had previous hospitalization for hemorrhagic fever with renal syndrome. Over one-third (38%) of participants encountered rodents or droppings in the past year in their homes or workplaces. Higher seroprevalence was found among office occupational workers than unemployed people (prevalence ratio [PR]:7.3, 95%CI: 1.3–53.5), and among those who lived near ponds than those who did not (PR:11.5, 95%CI: 1.6–54.7).

**Conclusion:**

Overall, the seroprevalence was low, but indicated some risk of infection among the adult population. Our results highlight potential occupational and residential risk factors for hantavirus infection in West Kazakhstan. Relevant public health interventions should include educating the population about promoting preventive practices, workplace hygiene, rodent control measures, and enhanced case diagnosis and management.

## 1 Introduction

Orthohantaviruses, also known as hantaviruses, are a group of zoonotic pathogens known for causing hantavirus infection in humans. These viruses predominantly transmit through contact with infected rodents and from inhalation of aerosolized viral particles from urine, droppings or saliva (1). In the Americas, hantavirus infection leads to hantavirus pulmonary syndrome, a severe cardiopulmonary disease (2). In Africa, Asia and Europe, it causes hemorrhagic fever with renal syndrome (HFRS) and nephropathia epidemica (NE) (3). Russia has the highest burden with an average of over 164,000 cases reported annually (4), followed by China which reports on average 10,000 cases annually (5). HFRS can be caused by Hantaan virus (HTNV), Amur virus, Seoul virus (SEOV), Dobrava-Belgrade virus (DOBV), or Puumala virus (PUUV) strains. Each has variable clinical presentations and a range of severity (6–8).

**Figure 1:**
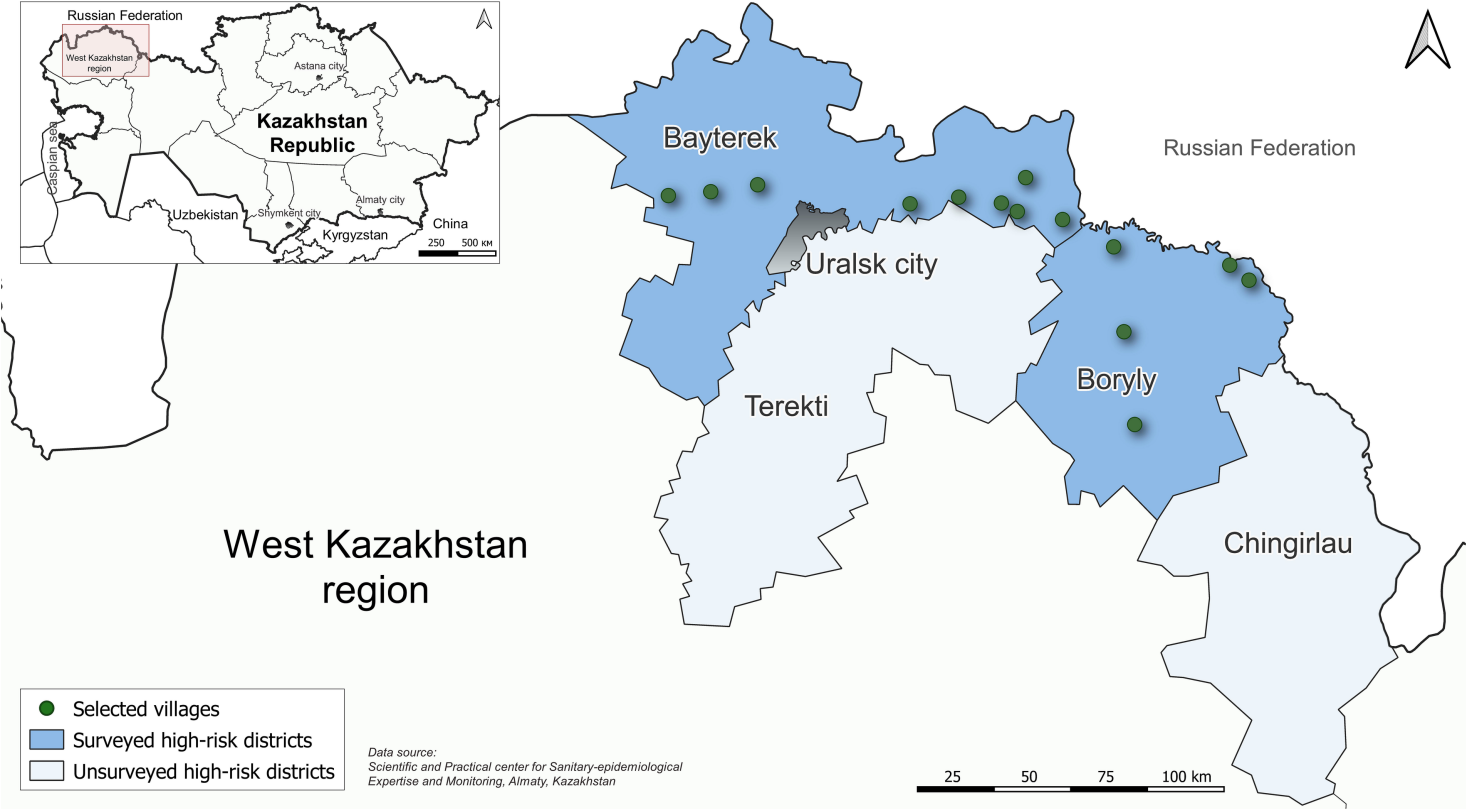
Surveyed study areas with location of selected villages in West Kazakhstan region, 2023.

Rural communities are usually at higher risk for HFRS than urban areas due to proximity to natural rodent habitats and often poorer sanitary conditions, which increase the likelihood of contact with rodents (9, 10). The population of the West Kazakhstan region is predominantly rural and is known to have increased risk for HFRS compared to other regions of Kazakhstan (11). The first human cases in West Kazakhstan were detected and serologically confirmed in 2000. By 2023, 251 cases had been confirmed in the region (Figure 1). West Kazakhstan region has a population of 683,327 and borders Russia near the Ural Mountains. The region has a unique habitat of flora and fauna, that may be favorable to species that can carry hantaviruses and other zoonotic infections (11, 12). Recent studies on the prevalence of antibodies to hantaviruses among host reservoirs (primary rodents) in Kazakhstan indicate that the virus is circulating in the areas previously considered free of them (13–15).

The healthcare system in the region includes district central hospitals and a regional infectious diseases hospital in the city of Uralsk. When healthcare workers suspect a person has HFRS, they are admitted to the regional infectious disease hospital for testing. Samples are sent to the Ural anti-plague station which is the only laboratory in West Kazakhstan with capacity to run HFRS ELISA IgM and IgG.

**Figure 2:**
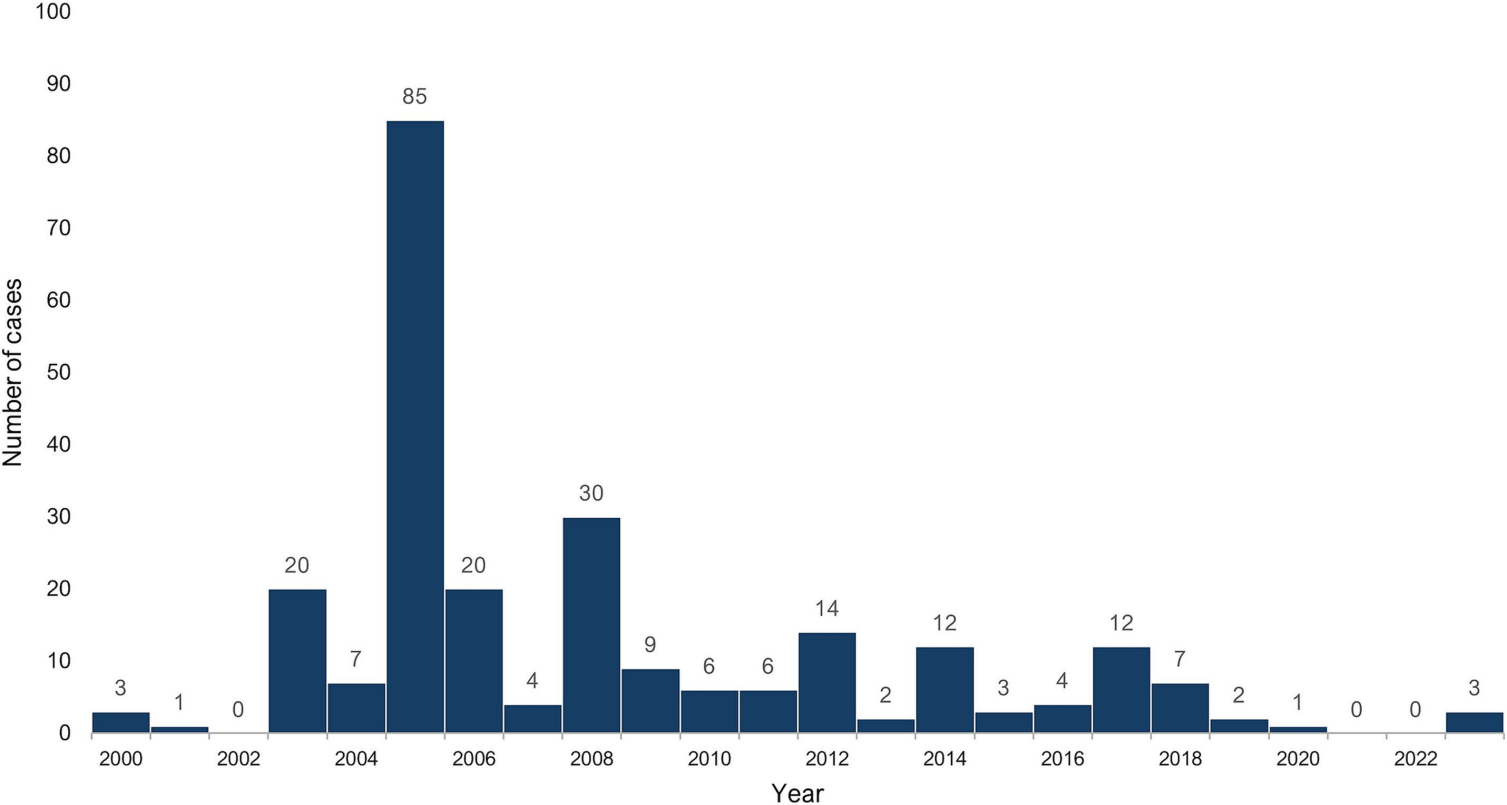
Registered cases of hemorrhagic fever with renal syndrome in Kazakhstan, 2000–2023.

In Kazakhstan the average incidence rate was 0.04 per 100,000 population over the past 20 years with the peak registered cases in 2005 due to the outbreak related to the increase in the number of rodents due to drought (Figure 2).

No confirmed HFRS cases were reported to the national surveillance system between 2020 and 2022, raising concerns about potential underreporting and delayed detection. The redirection of resources to deal with the COVID-19 pandemic may have resulted in decreased surveillance and reporting of other reportable diseases, including HFRS, during this period (16).

The goal of our study was to investigate the seroprevalence of hantavirus infection in West Kazakhstan to better understand the burden and identify possible risk factors to hantavirus infection in the region.

## 2 Methods

### 2.1 Study design

We conducted a cross-sectional seroprevalence study during June–July 2023 in 14 villages in Bayterek and Boryly districts in the West Kazakhstan region (Figure 1). These villages were selected because they were considered high-risk areas for hantavirus transmission over the past decade.

The study population included persons aged 18 years and older residing in villages in the two districts. Sample size was calculated by Kauermann et al. (17) method. We used systematic random sampling to select a minimum of 900 participants from public clinic registries. In Kazakhstan, all people living in a public clinic’s catchment population are registered at that clinic. Selected participants were recruited by telephone and asked to come for an appointment at their local clinic. To ensure inclusivity, particularly for individuals residing in remote areas, participants were invited to visit the centers at times convenient for them. A total of 921 individuals formed the final study population.

### 2.2 Data sources

#### 2.2.1 Survey tool

Trained nurses conducted face-to-face interviews. The questionnaire included questions about socio-demographic, clinical characteristics, and environmental and behavioral risk factors associated with hantavirus infection. Data entry was done using KoboToolBox (Kobo Organization, Cambridge, Massachusetts, USA) (18).

#### 2.2.2 Laboratory tests

We collected approximately 5 mL of whole blood from consenting participants. Samples were transported to the Ural anti-plague laboratory at +2°C to +4°C within 2–4 h of collection. There, samples were centrifuged, serum samples were aliquoted into two cryovials, and frozen at −20°C. Frozen samples were transported to the Especially Dangerous Diseases National Reference Laboratory in Almaty at −20°C within 72 h.

Serum samples were tested for the presence of IgG antibodies reactive to HTNV, DOBV, PUUV strains using enzyme-linked immunosorbent assay (ELISA) EUROLINE Anti-Hanta Profile 1 test kit^1^. Results were determined by assessing optical density (OD) using a microplate reader. Participants with OD ratios >0.8 were considered reactive. Positive serum samples were tested by Immunoblot Anti-Hanta Virus Pool 1 Eurasia test kit^2^ to identify the serotypes of hantaviruses. Bands with intensity from weak to very strong patterns were interpreted as positive.

The diagnostic test was performed and the results were interpreted according to the test-kit manufacturer’s instructions.

### 2.3 Ethical considerations

The study was approved by the Ethical Board of the National Center of Public Health, Ministry of Health of the Republic of Kazakhstan, under number 9/26.12.2022.

Written informed consent was obtained for all participants involved in the study. Children were not interviewed. Special considerations, if applicable, were addressed in accordance with ethical guidelines as approved by the Institutional Review Board (IRB) at the National Center of Public Health Care of The Ministry of Health of The Republic of Kazakhstan, based on international standards, including the Declaration of Helsinki and national regulations on biomedical research. Identifiable information and sensitive data were collected as necessary for research purposes, and safeguarded through encryption, restricted access, and secure storage protocols to ensure confidentiality and privacy.

### 2.4 Statistical analysis

Data cleaning and analysis was conducted in R version 4.3.1 statistical software. The questionnaire and laboratory databases were merged with Microsoft Excel using a unique participants’ identification number and then anonymized. Participants’ characteristics were summarized using descriptive statistics.

The outcome of interest was defined as the presence or absence of IgG antibodies reactive to hantavirus strains. The estimated seroprevalence and 95% confidence intervals were calculated for each group. Prevalence ratios (PR) and 95% confidence intervals (CI) were estimated by participant demographic, behavioral and environmental characteristics. The statistical significance of differences in prevalence between groups was determined using the χ2 and Fisher’s exact tests. After checking for multi-collinearity (phi coefficient), variables significant at *p* < 0.2 in bivariate analysis and known confounders (e.g., sex and age) were considered for multivariable Poisson regression.

## 3 Results

### 3.1 Recruitment flow

We selected 1,228 people from 14 villages in Boryly and Bayterek districts in West Kazakhstan region, of whom 971 (75.6%) agreed to participate and completed the questionnaire. Among those who consented, 921 had quality serum samples, and were included in the final study population.

### 3.2 Participant characteristics

Of the 921 participants 577 (63.0%) were female (Table 1). The median age was 53 years (standard deviation (SD): 16). And nearly one-half (48.0%) were aged 40–63 years. Among all participants 29.5% were retired, 27.7% were blue-collar workers, and 21.4% were unemployed. Most participants (72.0%) resided in single houses, 16.0% lived in houses with farm animals, and 12.0% in apartments.

**Table 1:** Sociodemographic and residential characteristics of study participants, West Kazakhstan region, 2023 (Total *N* = 921).

Characteristic	No. participants	%^a^
Sex		
Female	577	63.0%
Male	344	37.0%
Age groups, Median (SD)	53.0 (15.9)	
39 years and younger	242	26.0%
40–63 years old	440	48.0%
64 years and older	239	26.0%
Duration of residence in the villages		
Less than 3 years	61	6.6%
3 years and more	860	93.4%
District name		
Bayterek district	637	69.0%
Boryly district	284	31.0%
Education level^b^		
Upper level	252	27.0%
Lower level	669	73.0%
Occupation		
Retired	272	29.5%
Blue collar workers	255	27.7%
Unemployed	197	21.4%
Office workers^c^	88	9.6%
Service industry workers	74	8.0%
Housewife	35	3.8%
Income level		
More than 300$ per month	211	23.0%
300$ per month and less	710	77.0%
Marital status		
Single	273	30.0%
Married	648	70.0%
Housing type		
Apartment	111	12.0%
Homestead	146	16.0%
Single house	664	72.0%
Outdoor activities frequence		
Never	278	30.0%
Several times per month	211	23.0%
Several times per year	432	47.0%
Have forest areas within 500 meters of the house		
No	669	73.0%
Yes	252	27.0%
Have agricultural fields within 500 meters of residence		
No	833	90.4%
Yes	88	9.6%
Have a pond within 500 meters of residence		
No	913	99.1%
Yes	8	0.9%
Own domestic cats		
No	476	52.0%
Yes	445	48.0%
Own domestic poultry		
No	724	79.0%
Yes	197	21.0%
Own sheep, goats or cows		
No	726	79.0%
Yes	195	21.0%
Have a pantry (food storage room) in or near residence		
No	184	20.0%
Yes	737	80.0%
Frequency of housecleaning		
Never	57	6.2%
Several times per month	864	93.8%
Frequency of glove use during cleaning or gardening		
From time to time	312	34.0%
Most of the time	609	66.0%
Noticed rodents or their excreta from July 2022 to July 2023		
No	570	62.0%
Yes	351	38.0%
Noticed rodents or their excreta at home/household buildings, *n* = 351		
No	75	21.4%
Yes	276	78.6%
Noticed rodents or their excreta at work buildings, *n* = 351		
No	218	62.1%
Yes	133	37.9%
Was bitten by a rodent from July 2022 to July 2023		
No	918	99.7%
Yes	3	0.3%

a Column percent.

b Lower level (primary, lower secondary education), high level (upper secondary level education, any tertiary education).

c In the rural areas where the study was conducted, these “offices” are old 1–2 story structures, not modern business buildings, with poor sanitation conditions, and high likelihood of contact with rodents.

When asked about the environment/habitat within 500 meters of their residence, 27.0% of participants lived near a forest, 9.6% near agricultural fields, and 0.9% (*n* = 8) near ponds. Nearly half of participants (48.0%) owned domestic cats, and one of five (21.0%) owned domestic poultry or cattle animals (sheep, goats or cows). Household habits showed that 80.0% of respondents have a pantry,^3^ 93.8% clean their homes several times a month, and 66.0% use gloves most of the time during cleaning or gardening.

Rodent or rodent dropping sightings from July 2022 to July 2023 were reported by 38.0% (*n* = 351) of the participants. Of which (*n* = 351) the sightings near their home reported 78.6% and near their place of work reported 37.9%.

### 3.3 Seroprevalence and prior disease history

Hantavirus seroprevalence was 3.1% (*n* = 28, 95% Confidence interval (CI): 2.1–4.3). Of the 28 positives sera tested by Immunoblot, 14 had specific PUUV pattern,^4^ 7 had non-specific patterns, 6 had cross-reactivity pattern (PUUV, HTNV, DOBV). One serum showed a specific reaction for the HTNV and DOBV antigen.

When we compared those who tested positive for Hantavirus to those who did not, none of the 28 who tested positive had a history of HFRS, recent febrile illness, rash, or other clinical symptoms/signs consistent with HFRS. In contrast, 3 participants among whom tested negative reported a history of HFRS (Table 2).

**Table 2:** Clinical characteristic of study participants, West Kazakhstan region, 2023 (Total *N* = 921).

Characteristic	No. persons IgG positive/ no. tested	Seroprevalence (95% CI)	*p*-value^a^
Overall	21/893	3.1 (2.1–4.4)	
Hospitalization history with HFRS			0.992
No	28/918	3.1 (2.1–4.4)	
Yes	0/3	0.0 (0.0–69.0)	
Had any symptoms/complications from July 2022 to July 2023			0.999
No	26/828	3.1 (2.1–4.6)	
Yes	2/93	2.2 (0.4–8.7)	
Fever >101°F (38.3°C)			0.986
No	28/878	3.2 (2.2–4.6)	
Yes	0/43	0.0 (0.0–10.0)	
Lower back pain			0.989
No	28/899	3.1 (2.1–4.5)	
Yes	0/22	0.0 (0.0–18.0)	
Chills/shivers			0.272
No	27/910	3.0 (2.0–4.3)	
Yes	1/11	9.1 (0.4–43.0)	
Eye inflammation			**0.036**
No	27/917	2.9 (2.0–4.3)	
Yes	1/4	25.0 (1.3–78.0)	
Rashes			0.993
No	28/920	3.0 (2.1–4.4)	
Yes	0/1	0.0 (0.0–95.0)	
Acute renal failure			0.988
No	28/915	3.1 (2.1–4.5)	
Yes	0/6	0.0 (0.0–48.0)	
Low blood pressure			0.749
No	27/897	3.0 (2.0–4.4)	
Yes	1/24	4.2 (0.2–23.0)	

CI, Confidence interval. HFRS, Hemorrhagic fever with renal syndrome.

^aPearson’s Chi-squared test; Fisher’s exact test. Bolded variables are those with p-value <0.05.^

### 3.4 Seroprevalence and risk factors

Hantavirus seroprevalence did not statistically differ by sex (3.1% among females and 2.9% among males) (Table 3). The seroprevalence ranged from 2.3% among participants aged 40–63 years, 3.3% among 39 and younger and to 4.2% among those aged 64 and older but did not differ statistically by age. Office space workers^5^ had the highest seroprevalence among the different types of employment status, with 4.5%.

**Table 3:** Variables associated with hantavirus seroprevalence, West Kazakhstan, 2023 (Total *N* = 921).

Characteristic	No. persons IgG positive/ no. tested	Seroprevalence (95% CI)	PR (95% CI)	*P*-value	aPR (95% CI)	*P*-value
Sex						
Female	18/577	3.1 (1.9—5)	Reference		Reference	
Male	10/344	2.9 (1.5—5.5)	0.9 (0.4—2.0)	0.858	0.7 (0.3—1.8)	0.593
Age groups						
39 years and younger	8/242	3.3 (1.5—6.7)	Reference		Reference	
40–63 years old	10/440	2.3 (1.2—4.3)	0.7 (0.3—1.8)	0.429	0.7 (0.2—1.8)	0.391
64 years and older	10/239	4.2 (2.1—7.8)	1.3 (0.5—3.3)	0.619	2.2 (0.5—9.0)	0.289
District name						
Bayterek district	14/637	2.2 (1.1—5.2)	Reference			
Boryly district	14/284	**4.9 (2.8—8.3)**	**2.2 (1.1—4.6)**	**0.033**	**2.2 (0.93—5.02)**	**0.071**
Education level						
High education	7/252	2.8 (1.2—5.9)	Reference			
Incomplete or secondary education	21/669	3.1 (2.0—4.8)	1.1 (0.5—2.9)	0.779		
Occupation						
Unemployed	2/197	1.0 (0.2—4.0)	Reference		Reference	
Housewife	1/35	2.9 (0.2—17.0)	2.7 (0.2—29.4)	0.794	1.8 (0.1—20.2)	0.599
Retired	9/272	3.3 (1.6—6.4)	3.2 (0.7—14.4)	0.201	1.7 (0.3—13.4)	0.589
Blue collar workers	9/255	**3.5 (1.7—6.8)**	**3.4 (0.7—15.4)**	**0.167**	**4.1 (1.0—27.5)**	**0.079**
Service industry workers	3/74	4.1 (1.1—12.0)	3.8 (0.7—22.5)	0.273	**5.2 (0.8—41.5)**	**0.081**
Office workers^c^	4/88	**4.5 (1.5—12.0)**	**4.3 (0.8—22.9)**	**0.167**	**7.3 (1.3—53.5)**	**0.029**
Income level						
More than 300$ per month	6/211	2.8 (1.2—6.4)	Reference			
300$ per month and less	22/710	3.1 (2.0—4.7)	1.1 (0.5—2.9)	0.852		
Marital status						
Single	9/273	3.3 (1.6—6.4)	Reference			
Married	19/648	2.9 (1.8—4.6)	0.9 (0.4—2.1)	0.772		
Housing type						
Apartment	2/111	1.8 (0.3—7.0)	Reference			
Homestead	5/146	3.4 (1.3—8.2)	1.9 (0.4—13.3)	0.443		
Single house	21/664	3.2 (2.0—4.9)	1.8 (0.5—11.0)	0.447		
Outdoor activities frequence (camping, fishing, wild swimming)						
Never	12/278	4.3 (2.4—7.6)	Reference		Reference	
Several times per year	16/643	**2.5 (1.5—4.0)**	**0.6 (0.3—1.3)**	**0.185**	**0.4 (0.2—1.0)**	**0.051**
Have forest areas within 500 meters of the house						
No	20/669	3.0 (1.9—4.7)	Reference			
Yes	8/252	3.2 (1.5—6.4)	1.1 (0.45—2.3)	0.886		
Have agricultural fields within 500 meters of residence^a^						
No	23/833	2.8 (1.8—4.2)	Reference		Reference	
Yes	5/88	5.7 (2.1—13.0)	2.1 (0.7—4.9)	0.144	1.4 (0.4—3.9)	0.587
Have a pond within 500 meters of residence^a^						
No	26/913	2.8 (1.9—4.2)	Reference		Reference	
Yes	2/8	**25.0 (4.5—64.0)**	**8.8 (1.4—29.3)**	**0.003**	**11.5 (1.6—54.7)**	**0.005**
Own domestic cats						
No	11/476	2.3 (1.2—4.2)	Reference		Reference	
Yes	17/445	3.8 (2.3—6.2)	1.7 (0.8—3.6)	0.194	1.6 (0.7—3.6)	0.295
Own domestic poultry						
No	20/724	2.8 (1.7—4.3)	Reference			
Yes	8/197	4.1 (1.9—8.1)	1.5 (0.6—3.2)	0.357		
Own sheep, goats or cows^a^						
No	19/726	2.6 (1.6—4.1)	Reference		Reference	
Yes	9/195	4.6 (2.3—8.9)	1.8 (0.8—3.8)	0.161	1.6(0.6—3.9)	0.292
Have a pantry (food storage room) in or near residence						
No	3/184	1.6 (0.4—5.1)	Reference			
Yes	25/737	3.4 (2.3—5.0)	2.1 (0.7—8.7)	0.231		
Frequency of housecleaning						
Never	1/57	1.8 (0.1—11.0)	Reference			
Several times per month	27/864	3.1 (2.1—4.5)	1.8 (0.2—12.7)	0.646		
Frequency of glove use during cleaning or gardening^a^						
From time to time	14/312	4.5 (2.6—7.6)	Reference		Reference	
Most of the time	14/609	2.3 (1.3—3.9)	**0.5 (0.3—1.1)**	**0.077**	**0.5 (0.2—1.1)**	**0.085**
Noticed rodents or their excreta from August 2022 to July 2023^b^						
No	15/570	2.6 (1.5—4.4)	Reference		Reference	
Yes	13/351	3.7 (2.1—6.4)	1.4 (0.7—2.9)	0.367	1.5 (0.7—3.4)	0.293
Noticed rodents or their excreta at home/household buildings, *n* = 351						
No	3/75	4.0 (0.9—11.6)	Reference			
Yes	10/276	3.6 (1.9—6.8)	0.9 (0.2—3.2)	0.844		
Noticed rodents or their excreta at work buildings, *n* = 351						
No	7/218	3.2 (1.8—9.7)	Reference			
Yes	6/133	4.5 (1.8—10.0)	1.4 (0.5—4.1)	0.541		

PR, Prevalence ratio. Calculated using bivariate analysis with hantavirus seropositivity (yes/no) as the outcome and the variable on the left as the exposure. aPR, Adjusted prevalence ratio. Calculated using multivariable Poisson regression model with hantavirus seropositivity (yes/no) as the outcome and the variables significant at *p* < 0.2 in bivariate analysis, or known confounders on the left as the exposure. CI, Confidence interval. Bolded variables are those with *p*-value < 0.10.

a Included in the final multivariable model due to *p*-value < 0.2 was used as cutt-off point.

b Included in the final multivariable model as it better reflects the epidemiologic risk factors.

c In the rural areas where the study was conducted, these “offices” are old 1–2 story structures, not modern business buildings, with poor sanitation conditions, and high likelihood of contact with rodents.

Participants who reported infrequent use of gloves while gardening had seroprevalence of 4.5% (95% CI: 2.6–7.6), compared to those who used gloves most of the time, 2.3% (95% CI: 1.3–3.9). The prevalence among participants living near ponds was 25.0% (n = 2/8, 95% CI: 4.5–64.0).

There was no significant difference in seroprevalence among those who had noticed rodent activity or droppings in their homes or workplaces in the preceding year compared to those that did not observe rodent activity (*p* > 0.05).

In multivariable Poisson regression, occupation was significantly associated with seropositivity. Specifically, office space workers (PR = 7.3, 95%CI 1.3–53.5, *p* = 0.029) had increased risk compared to those who are unemployed. Risk was also increased for people who lived near ponds (PR = 11.5, 95%CI 1.6–54.7, *p* = 0.005) compared to those who did not live near a pond.

Risk was lower for people who regularly used gloves while gardening compared to those who not (PR = 0.4, 95% CI 0.2–1.0), though not significant at (*p* < 0.05).

## 4 Discussion

Prior to our investigation, the seroprevalence of hantavirus infection among people living in West Kazakhstan was unknown. We found that approximately 3 in 100 adults had serologic evidence of exposure to hantavirus. Considering the Euroimmun test employed in our study, it is possible that our seroprevalence estimate is slightly conservative, as the test may not detect all true positive cases. However, in several studies which used the Euroimmun test for detecting IgG antibodies to hantaviruses demonstrated higher sensitivity and specificity (19, 20). Even with this potential underestimation, our findings suggest that the current passive case reporting surveillance system for hantaviruses likely underestimates true disease burden given that only 251 cases have ever been reported in this region with nearly 800,000 inhabitants.

Other important consideration is using the test pool sensitive to PUUV, HTNV and DOBV hantavirus strains. Although Tula virus (TULV) has been detected in West Kazakhstan, as documented by Tukhanova et al. (14), historically, PUUV has been the primary hantavirus circulating in West Kazakhstan, with its presence well-documented in routine surveillance. Yet the relatively low number of reported human TULV infections was registered in the region, the focus of our study was on clinically significant strains, as they pose a higher risk of severe disease (6).

Additionally, population mobility may further influence the observed seroprevalence. Seasonal labor migration, particularly among men traveling to neighboring regions for work in the oil industry, followed by engagement in manual labor upon their return, may increase exposure to hantavirus (21). These migratory patterns could result in the exclusion of higher-risk individuals from our sample, potentially contributing to the underestimation of seroprevalence.

Our findings are consistent with typical patterns of hantavirus epidemiology (22). Higher seroprevalence was noted among residents near agricultural fields and ponds that are essential for rodents’ life cycles, which concise with other studies (23, 24). People who were seropositive lived in areas that are common habitats for the common vole – the main reservoir of infection in the region (11).

Participants reported high frequency of household practices and animal ownership that are associated with hantavirus exposure. However, we did not detect a significant association of these with hantavirus seroprevalence. A study by Wang et al. (25) found that infrequent human activity in poorly ventilated spare rooms may facilitate rodent reproduction, increasing the risk of hantavirus transmission through inhalation of infected aerosols. Some studies (9, 26) linked food contamination by rat excreta to increased risk, emphasizing the importance of proper food handling and storage practices. People can also become infected with hantavirus by touching their mouth or nose after handling contaminated materials. In our study, we did observe lower hantavirus seroprevalence among people who reported always using gloves when cleaning or gardening compared to those who did not, though this difference was not significant.

Among participants that tested positive, none reported prior hantavirus diagnosis, and none recalled having had symptoms consistent with hantavirus, suggesting a predominance of milder forms of the disease (3). A study in West Kazakhstan region found that individuals at risk of multiple or repeat infections, may develop immunity, which could result in mild or asymptomatic hantavirus infection (11). The lack of symptoms among IgG positive participants could also be explained by persistence of antibodies for up to a year or more after past illness (27, 28). It is important to note that PUUV infection, identified through immunoblot testing, usually results in mild illness with spontaneous full recovery. However, while most patients experience full recovery of renal function, there is a risk of delayed development of renal complications, including chronic renal impairment (11, 29–31).

Unexpectedly, our study identified higher prevalence of hantavirus antibodies among individuals reporting limited outdoor activities compared to those who reported outdoor activities and those engaged in office occupational roles versus unemployed. While it is expected that farm workers will have higher seroprevalence rates than the other groups (25, 32, 33), our results indicate diverse activities or behaviors among office workers in West Kazakhstan region. They may encounter hantavirus-carrying rodents potentially in and around their workplaces (34, 35), with the likely exposure factor being enclosed (office building) spaces where contamination with viral particle aerosols is higher (36). While we did not specifically ask about the sanitary conditions in their workplaces, it is known that in the rural areas where the study was conducted, these “offices” are old 1–2 story structures, with often poor sanitation conditions, and increase the likelihood of contact with rodents. This finding may be an outlier but warrants further consideration and study.

Our study was subject to several limitations. First, the study observed low participation among men due to seasonal work, who may be at greater risk. Since the response rate was 75.6%, participation bias may have affected our results. Second, our study was underpowered to detect differences between groups at seroprevalence of 3.1%. Third, our findings were potentially subject to recall bias because participants may fail to remember specific exposures or mild symptoms. Fourth, the seroprevalence estimate in our study may be slightly underestimated due to the sensitivity (88.2%) and specificity (94.1%) of the Euroimmun test used. The inability to detect all true positive cases remains a limitation and could contribute to a conservative estimate of seroprevalence.

To reduce the effect of these limitations, systematic random sampling was employed, enhancing the reliability and validity of the study findings by reducing selection bias. We also used local family nurses for the recruitment process leveraging their frequent contact and trust in the community, which likely increased engagement in the study. In addition, we cross-referenced reported symptoms with medical records when possible. While our test was not the same used routinely in the country by public health authorities, the Euroimmun test we used has documented high sensitivity and specificity, giving us confidence in our results. The study encompassed two endemic districts in the West Kazakhstan region, providing a broad and representative understanding of the hantavirus seroprevalence in these key areas.

## 5 Conclusion

In conclusion, we found small proportion of adults in rural West Kazakhstan have evidence of exposure to hantavirus. We identified potential occupational and environmental exposures for hantavirus infection. We also identified the high prevalence of rodent activity in people’s homes and places of work. Poor housing conditions, especially in rural areas, were also found to contribute to higher infection risk. These findings emphasize the need to improve rodent control in the workplaces, home and surrounding habitats to mitigate the risk of hantavirus transmission. Public health interventions should focus on educating the public about the risks of rodent exposure and promoting preventive practices and sanitation.

Our study also highlights that reported cases likely underestimate the true incidence of infection and cases. This underscores the need to complement passive surveillance with periodic seroprevalence studies, including expanding the geographical coverage to additional districts, to gain a more accurate understanding of hantavirus circulation in the region. Establishing more laboratories equipped with capacity to conduct hantavirus testing, alongside educating healthcare providers can improve case detection.

Finally, migratory patterns and seasonal occupational mobility also potentially contribute to the underestimation of seroprevalence. Further studies that account for seasonal and occupational mobility are needed to better assess its impact on hantavirus exposure in the region. Given potential renal complications of hantavirus infection, we propose exploring the possibility of a targeted serosurvey among patients with chronic kidney disease compared to the general population. Observing higher hantavirus antibody prevalence in Chronic kidney disease (CKD) patients could provide insights into its potential contribution to CKD in the region. Understanding hantavirus transmission dynamics and addressing local risk factors through tailored public health measures are crucial for reducing infection risks and improving health outcomes in affected populations.

## Data Availability

The datasets presented in this article are not readily available because the data supporting the findings of this study are restricted due to Kazakhstan national legislation rules pertaining to especially dangerous infections. Official requests for the data can be made on request to the government of Kazakhstan. Requests to access the datasets should be directed to corresponding author, Ulyana Gubareva, ulyana.9355@gmail.com.

## Footnotes

^1^EUROLINE Anti-Hanta Profile 1 (IgG) (EUROIMMUN AG, Lübeck, Germany) test kit has previously been found to be sensitive (100%) and specific (100%) in study with 157 pre-characterized patient samples; and to be sensitive (88.2%) and specific (94.1%) in a study with thirty-nine pre-characterized patient samples. Borderline results were not included in the calculation.

^2^Anti-Hanta Virus Pool 1 “Eurasia” ELISA (EUROIMMUN AG, Lübeck, Germany) test sensitivity and specificity amounted to 92.5 and 88.9%, respectively.

^3^Pantry - a room or closet in which food, groceries, and other provisions, or silverware, dishes, etc., are kept.

^4^By comparing intensities of color reaction of sample band to control band.

^5^In the rural areas where the study was conducted, these “offices” are old 1–2 story structures, not modern business buildings, with poor sanitation conditions, and high likelihood of contact with rodents.
